# 

**Published:** 1888-02

**Authors:** 


### TABLE OF CONTENTS.

ORIGINAL AND SELECTED ARTICLES.

Dislocation of the shoulder of long standing—a clinical lecture, by J. McF. Gaston, M. D., Atlanta, Ga. ... 41
Self-castration resorted to as a remedy for seminal emissions, reported by K. H. Boland, M. D., Atlanta, Ga ... 43
Recent progress in obstetrics and gynecology in Germany, by E. S. McKee, M. D, Cincinnati, Ohio ... 45
Recent advances in the treatment of consumption ... 50
On the treatment of piles by injection of carbolic acid, by Francis L. Haynes, M. D., Los Angeles, Cal ... 53
Electricity in post partum hemorrhage ... 56

ABSTRACTS AND GLEANINGS.

The enemata syringe—some suggestions as to its use and value ... 57
Amylene hydrate; strophanthus. ... 59
Methylene chloride compared with chloroform ... 60
Operative treatment of internal obstruction of the bowels ... 61
Venesection in pneumonia ... 62
Intestinal obstructions; a case of poisoning with cocaine ... 63
Galvanism in the treatment of indolent ulcers; cocaine for neuralgia ... 64
Treatment of rheumatism in the Jefferson College Hospital; tobacco amblyopia ... 65
Amylene Hydrate; sore nipples ... 66
Decoction of white oak bark in internal hemorrhoids; rabies from tanacetic acid; the Texas supreme court ... 67
Treatment of dysentery in Texas; antipyretics 68
Avulsion of ingrowing nail under hypnotism; painless destruction of nævi ... 69
Pyridin in asthma; castration in epilepsy; a New York wine merchant; guaiac tinct. in acute tonsillitis ... 70

SCIENTIFIC ITEMS.

Artificial silk; a new gas; do horses have horse sense ... 71
Changes in teeth ... 72

PRACTICAL NOTES AND FORMULÆ.

Asthma; for chronic catarrh; for stomatitis; nasal catarrh ... 73
Salicylic plaster comp; muriate of ammonia. 74

EDITORIALS AND MISCELLANEOUS.

Editorial brevities ... 75
The American Medical Association; contempt of court ... 76
Ogeechee Association; antiseptic treatment of intestinal affections ... 77
Parke, Davis & Co.; books and pamphlets received ... 78
Receipted ... 79
Special notes ... 80

